# Evaluating the impact of patient and carer involvement in suicide and self‐harm research: A mixed‐methods, longitudinal study protocol

**DOI:** 10.1111/hex.13000

**Published:** 2019-12-05

**Authors:** Donna L. Littlewood, Leah Quinlivan, Sarah Steeg, Carole Bennett, Harriet Bickley, Cathryn Rodway, Roger T. Webb, Navneet Kapur

**Affiliations:** ^1^ NIHR Greater Manchester Patient Safety Translational Research Centre The University of Manchester Manchester UK; ^2^ Centre for Mental Health and Safety School of Health Sciences The University of Manchester Manchester UK; ^3^ Manchester Academic Health Science Centre The University of Manchester Manchester UK; ^4^ Greater Manchester Mental Health NHS Foundation Trust Manchester UK

**Keywords:** evaluation, mental health, patient and public involvement, self‐harm, suicidal behaviour

## Abstract

**Background:**

Patient and public involvement (PPI) is becoming more commonplace in mental health research. There are strong moral and ethical arguments for good quality PPI. Few studies have documented and evaluated PPI in self‐harm and suicide research. Inconsistent reporting of PPI makes it difficult to discern practices that deliver quality, effective and meaningful involvement. It is important to understand and address emotional support needs of PPI members contributing to sensitive topics such as suicide and self‐harm. Therefore, this study will examine the effect of PPI on self‐harm and suicide research and explore patients', carers' and researchers' experiences and views in relation to the quality of PPI practice and provision of appropriate support for PPI members.

**Methods:**

This protocol outlines the longitudinal, mixed methodological approach that will be taken. Qualitative and quantitative data will be collected via baseline and repeated questionnaires, document review and semi‐structured interviews. Both PPI members and researchers will be invited to participate in this study. The two‐year data collection period will enable evaluation of PPI throughout the entire research cycle. An integrated approach will be taken to data analysis, using inductive thematic analysis and descriptive and repeated measures analyses, to address specified study aims.

**Dissemination:**

Findings from this study will inform practical guidance to support self‐harm and suicide researchers in effectively involving people with experiential knowledge in their research. Analyses will offer insight into the effect of PPI throughout the research process and assess changes in PPI members' and researchers' experiences of involvement across a two‐year period.

## 1 INTRODUCTION

There are strong moral and ethical arguments for involving people with experiential knowledge in research, which recognize that people should be involved as leaders or partners in research which may impact them.^1^, ^2^ It therefore follows that patients and the public should be provided opportunities to be involved in research irrespective of whether such involvement translates into a positive effect on the ensuing research. That said, systematic reporting and evaluation of patient and public involvement (PPI) is vital to understand what works well generically and in the specific context of particular research topics. Effective PPI is of clear benefit to research,^3^, ^4^ PPI members^5^, ^6^ and researcher(s).^7^ Yet, reporting of PPI remains inconsistent.^8^ The development of this evidence base will provide best practice guidance to improve the quality of PPI in future research.^8^ In seeking to address this issue of inconsistent reporting, Staniszewska and colleagues^8^, ^9^ have developed a reporting checklist, the Guidance for Reporting Involvement of Patients and the Public (GRIPP; GRIPP2). The GRIPP2 checklist^8^ calls for researchers to critically reflect and share their insight into PPI practice by reporting both the positive and negative impact of PPI on (a) the research, (b) the PPI members and (c) the researchers. Furthermore, the checklist highlights the importance of considering how specific contextual factors may have acted as a barrier to, or facilitated, effective PPI. However, impact or the effects of PPI should not be examined without considering the quality of PPI.^10^ In the UK, good quality PPI has been described as part of the National Standards for Public Involvement in Research^11^ and a wealth of resources on developing a good quality PPI approach are available via the national advisory organization, INVOLVE. 12

Research groups working within the field of suicide and self‐harm are actively involving people with experiential knowledge in their studies.^13^, ^14^, ^15^, ^16^ However, there is a dearth of published evaluations of PPI in this specialized research field. To our knowledge, there are just two evaluations in the existing literature. First, Awenat and colleagues^5^ conducted a qualitative investigation that focussed solely on the experiences of ex‐prisoners who were members of a PPI group for research examining suicide prevention in prisons. Members described how their involvement in research helped them to develop more positive self‐perceptions. Findings from this study have important implications for researchers who plan to incorporate PPI into their research. For instance, developing positive relationships between the researchers and PPI members was seen as being vital to the sustained engagement of the PPI group. Furthermore, honorary contracts were provided to PPI members to provide access to university resources, such as the library. For some group members, this formal association with the university also helped them to feel valued and accepted.

Second, Maclean and colleagues'^17^ recent commentary on PPI in their research investigating therapy for men who attended hospital emergency department following self‐harm described how they tailored their PPI practice to meet group members' needs. By developing flexible processes, they were able to support members' mental well‐being; enabling them to take breaks and return to the group, as and when appropriate. They also described how they reserved time at meetings for members to ‘offer testament’ by sharing their personal experience in relation to the research topic. Both of these publications^5^, ^17^ provide useful insight for researchers developing plans to involve people with experiential knowledge in their research. They also illustrate that, whilst many of the principles and practices of good PPI will apply irrespective of the research topic, there can be particular factors that are specific to the research setting or context.

One such factor in the field of suicide and self‐harm research is ‘safety’—specifically, ‘how can people with experiential knowledge be *safely* involved?’ PPI members are commonly asked to draw upon their own experiences when contributing to research projects. In the specific context of suicide and self‐harm research, it is possible that reflecting on such experiences may consequently cause the individual to feel upset or distressed. The potential for suicide and self‐harm research to do harm has been examined within the context of research participation. Findings from a meta‐analysis of 18 studies suggest that participation in suicide‐related research is generally not associated with increased levels of distress or suicidal thoughts.^18^ Furthermore, participation is generally associated with more positive outcomes, with recent follow‐up data indicating that for some people, these positive outcomes are sustained into the long‐term.^19^ However, there are important differences between involvement in research as a participant in comparison with involvement as a PPI member. For instance, research procedures are subjected to scrutiny during ethical review which includes the assessment of the likelihood of risk to research participants. In contrast, PPI processes are not routinely subjected to the same formal review process. Although researchers may be expected to embed the same ethical approaches when involving PPI members in their work, the practicalities of involvement are arguably more complex. For example, consider a group of researchers involving a PPI group to develop a topic guide for their study about the methods people use to harm themselves with. Here, part of the PPI members' role is to judge the extent to which they view the topic guide as acceptable, sensitive and appropriate for use with people who have similar lived experience. In this task, the researchers are exposing PPI members to material that they may find upsetting, distressing and subsequently deem to be inappropriate for use. This potential risk was acknowledged in a recent survey of early career researchers, working in the field of suicide, self‐harm and mental health research.^20^ That said, although there are challenges to involving people with lived experience in suicide and self‐harm research, it could be considered immoral to exclude PPI and also lead to poorly designed research studies with inappropriate methods and materials and ineffective recruitment strategies.^21^ It is important for researchers to consider this possibility and ensure they are able to provide appropriate emotional support to their PPI partners if such challenging circumstances arise. To our knowledge, there is no published guidance on meeting emotional support needs of PPI members in suicide/self‐harm research. Clinically trained mental health researchers have utilized their nursing skills to address members' emotional needs and develop a safe and supportive PPI environment.^6^ However, it is also important to establish and maintain boundaries, by providing support specifically within the context of the research that is being conducted.^22^ For example, in previous research, PPI members have provided the research team with information about their personal support networks or have developed a crisis plan for implementation should they become distressed in the course of their involvement with research.^22^ Minimizing risks to members of PPI groups should be a key component of PPI plans. However, researchers need to balance their commitment to ‘do no harm’ whilst avoiding paternalistic practice and ensuring the individual retains the power to make informed choices regarding involvement in the PPI group.

### 1.1 Aims

In lieu of any systematic evaluation of the quality of PPI practice and the effect that it may have on suicide and self‐harm research, the planned study has three main aims:

1. To understand how PPI over the two‐year research programme effects: (a) the research; (b) the PPI members and (c) the researchers.
2. To understand what constitutes quality, effective and meaningful involvement in sensitive research and the practice perceived to facilitate and hinder it.
3. To explore members' and researchers' views and experiences regarding optimal and appropriate support and safety procedures for PPI groups in suicide and self‐harm research.

## 2 METHODS

### 2.1 Study design

This longitudinal, mixed‐methods design study will comprise questionnaires, document review and semi‐structured interviews. A sequential mixed‐methods approach will be taken, whereby analysis of the data collected via the questionnaires will inform the subsequent semi‐structured interviews. As such, data collection will be multi‐staged across a two‐year programme of research to enable evaluation of involvement at different stages of the research cycle. In lieu of an available and established PPI group with relevant experiential knowledge, PPI in designing and developing this protocol is comprised of the following: (a) the inclusion of a public contributor as part of the research team who has both relevant lived experience and extensive experience as a member of a range of PPI groups, and (b) the involvement of an external, independent contributor (with experience of self‐harm and suicidal thoughts) who reviewed the initial research protocol and data collection tools. Input was also sought from an academic Lead for Patient and Public Involvement. The University of Manchester who reviewed and made suggested revisions to the initial protocol and data collection methods. Ethical approval for this study was granted by The University of Manchester Research Ethics Committee 3 (ref: 2019‐5360‐9169).

### 2.2 Establishment of the PPI panel

This current study will evaluate the involvement of the PPI panel for The Centre for Mental Health and Safety at The University of Manchester. The PPI panel was formed in November 2018. Prior to this, the research centre had created separate PPI panels for each new project. By establishing a single generic group for the research centre, we intend to build a sustainable community of people with lived experience of self‐harm and suicide who are actively involved in the research conducted by investigators in our Centre. Researchers in this group use a wide‐range of quantitative and qualitative methodologies.

There were three pre‐requisite criteria for PPI group membership: (a) personal experience of self‐harm, and/or of suicidal thoughts or attempts, and experience of accessing mental health services (patient members); or (b) experience of caring for someone with the lived experience of self‐harm, and/or of suicidal thoughts or attempts, and experience of accessing mental health services (carer members) and (c) aged 18 years and over. These criteria were established based on the research centre's current programme of research, which focusses on the role of adult mental health services in relation to self‐harm and suicide. We chose to include carer members within the group, in accordance with published literature that has highlighted the importance of family involvement in suicide prevention.^23^, ^24^, ^25^

### 2.3 Study participants

Eligibility for participation in this evaluation study will be restricted to the following: (a) members of the Centre for Mental Health and Safety PPI Panel; (b) researchers who have involved the panel in their work. All panel members will be sent an email or postal invitation to participate in the study, based on their personal communication preference. In order to ensure that PPI members do not feel obligated to participate in the evaluation study, the email invitation will stress that participation is not obligatory and that participation will be on an entirely voluntary basis (unlike PPI activities for which the group are remunerated). It will also be made clear that membership as part of the research advisory panel will not be affected if they choose not to take part in the proposed evaluation study. Should they express an interest in participating, they will then be sent a participant information sheet which provides a more in‐depth overview of the study; that is what participation will entail, how data will be collected, analysed and stored, confidentiality, and research governance processes and procedures. There are currently 14 PPI members who contribute at meetings and remotely, and a further six who contribute solely via remote methods. Any additional individuals who join the PPI group during the evaluation period will also be invited to participate in the study.

All researchers who seek to involve the panel in their work will be sent a separate email to invite them to participate in this evaluation study. It is difficult to estimate the likely sample size of researcher participants. However, within the centre there are currently 20 researchers, including research assistants, PhD researchers, post‐doctoral researchers and principal investigators. Researchers in the wider University of Manchester network may also request involvement from the panel. If they are interested, then a researcher‐specific participant sheet will be sent for their consideration.

In seeking to publish findings from this evaluation study, it will not be possible to maintain anonymity of the research centre or PPI group involved in the study. This will be made clear to those individuals who choose to participate in this study. To protect anonymity, all participants will be assigned a unique identifier on enrolment to the study. The first author (DLL) will hold the identifying information in order to link questionnaires and interview data. Further, we will give all participants the option to review quotations included in the resulting manuscript, prior to submission for publication.

Retention of participants is a common challenge faced in longitudinal studies. In the current study, we seek to mitigate this challenge by tailoring data collection methods to meet participants' preferences (eg providing options of internet or postal questionnaire submissions) and also maintain regular contact with participants throughout the data collection period.^26^

### 2.4 Data collection

#### 2.4.1 Panel member baseline questionnaire

On entry to the evaluation study, all panel participants will be asked to complete a baseline, background questionnaire. The nine‐item questionnaire which consists of fixed‐ and open‐choice questions, will cover demographical information, experience of suicidal thoughts and/or self‐harm, previous experience of research and initial expectations regarding their involvement in the PPI panel (eg How do you expect to be involved in supporting this research?; What do you hope to gain from your involvement?).

#### 2.4.2 Panel member repeated questionnaire

A repeated questionnaire will be administered to examine panel members' current experience of involvement and ideas as to how this could be improved. Specifically, items will assess type of involvement, how it changed research, extent to which involvement was perceived to have a major or minor impact on research and generally how satisfied they are with their involvement (eg To what extent do you feel empowered by your involvement role?). Additional questions will assess beliefs regarding the impact of involvement on members' well‐being. (eg Did taking part in this involvement activity have any effect on your current mood?). This questionnaire is an adaptation of a previous tool used to evaluate the quality of involvement in research and how quality changes over time.^27^ The 11‐item questionnaire uses a combination of free‐text response and fixed‐choice questions, which includes the use of nine‐point Likert scales. Panel participants will be asked to complete the current experiences questionnaire within one week of each involvement activity (ie meeting or remote task) occurring between March 2019 and March 2021. Whilst the number of repeated assessments may vary due to differing levels of panel involvement, we anticipate that questionnaire data will be collected approximately four to five times per year. Questionnaires will be provided electronically via a secure internet‐based survey, or in paper format, based on participants’ preferences.

#### 2.4.3 Researcher experience questionnaire

The researcher questionnaire was designed to assess researchers’ previous experiences of involving lived experience groups, and their views on the impact that involving this panel had on them (ie their well‐being and competency as a researcher) and their research (ie summary of any changes to research, any difficulties in implementing changes, and extent to which involvement was perceived to have a major or minor impact on research). The questionnaire was developed by members of the research team (DLL, LQ, SS). The questionnaire uses a combination of fixed‐choice (nine‐point Likert scale) and free‐text responses (eg Can you give any examples on how PPI has impacted your ability as a researcher?). Questionnaires will be administered via email, within one week of the involvement activity (eg attendance at a meeting to discuss a specific task or process with the group, or remote feedback on research documents). Researchers will be invited to complete the questionnaire each time they involve the PPI group in their research. Consequently, the number of repeated assessments will vary due to differing levels of involvement.

#### 2.4.4 Document review

Throughout the two‐year evaluation period, members of the research team (DLL, LQ, SS) will review relevant documents to extract and record examples and outcomes to illustrate the extent to which involvement activities had (a) any effect, (b) no effect on the Centre's research or (c) is likely to have an effect in the future. Here, we will assess what changes (if any) were made to the research, whether there were any barriers to implementing these changes, and evidence of resulting effect of any changes. For example, the PPI group may be asked to help develop a recruitment strategy for a new study. We will record suggestions made from the group, and the number of participants recruited following their suggestions. We will review all research documents that the PPI panel are asked to review, actions and minutes from panel meetings, and reporting of PPI for funding bodies. Further, any participant or ethical panel feedback which relates to the involvement of the panel will also be included.

#### 2.4.5 Semi‐structured interviews

Interviews with participants will be conducted by an independent researcher at the end of the evaluation period (March to August 2021). The interviews will explore participants' reflections on (a) their involvement across the research cycle, (b) their views on what constitutes good quality and meaningful involvement and practices that facilitate and hinder it, and (c) what steps researchers can take to effectively and safely involve people with lived experience. Findings from the repeated questionnaires and document review will be used to inform the development of the interview topic guide. Furthermore, the topic guide will contain questions to seek views on the quality of PPI by asking participants to reflect on each of the six National Standards for Public Involvement in Research.^11^ Consequently, the interviews will build a more in‐depth understanding of the topics and themes raised in the earlier stages of data collection. All interviews will be audio‐recorded and fully transcribed. To protect participant confidentiality, all identifiable data (including names of family, friends, participant, other PPI members, researchers and places) will be removed at the point of transcription.

### 2.5 Data analysis

A sequential approach will be taken to data analysis whereby quantitative and qualitative data collected via the questionnaires will be analysed first and used to inform the subsequent semi‐structured interviews. Descriptive statistics will be generated by analysis of the data collected via the questionnaires. Within‐subject, repeated measures Wilcoxon signed rank tests will be applied to assess changes in scores on the repeated panel member questionnaires. Scores on the fixed‐choice questions will be compared between various time points and over the course of the evaluation to examine perceived short‐term and long‐term effects experienced by participating panel members, addressing our first aim. Our main outcome measures will be panel members' rating of ‘To what extent, do you feel able to make a contribution to the research conducted by the University of Manchester?' Secondary outcomes will include feelings of empowerment, satisfaction and well‐being.

Where repeated measures are available, scores for researchers involved in the study will also be examined to address the first study aim. Here, outcomes will include perceptions of PPI impact on research, researcher well‐being and competency. A sample size of 30 or more is required to detect associations of weaker strength, although stronger associations can be detected by examining samples of 12‐15 participants.^28^ These data will be analysed by the third author (SS) using SPSS.

Qualitative data collected by questionnaires and interviews will be stored, managed and analysed within NVivo. An inductive thematic analysis, informed by the work of Braun and Clarke,^29^ will be performed. An integrated and constant comparative analytical approach^30^ will be taken, whereby an initial analysis of the data collected via the questionnaires and document review will inform the subsequent semi‐structured interviews. Specifically, three members of the research team (DLL, LQ, CB) will read and familiarize themselves with the qualitative questionnaire data and extracted data from the document review. They will individually conduct initial coding to address all three of the specified aims. The coders will then present these initial codes for group discussion with the independent researcher, with a view to highlighting specific areas to be explored further through the semi‐structured interviews.

Interview data will be incorporated into the existing dataset and analysed thereafter by the four researchers and an independent qualitative researcher. In brief, the researchers will take a constant comparative approach; code transcripts independently, then discuss and refine the emerging coding framework, before returning to the data to conduct further cycles of coding and refinement. The final thematic structure will be developed through group discussion to reach a consensus.

The four researchers conducting the qualitative analysis possess different backgrounds: two self‐harm and suicide researchers (DLL, LQ), an independent PPI contributor with relevant experiential knowledge (CB) and an independent qualitative researcher. This combination of expertise will help to increase the trustworthiness of the analysis.^31^ Furthermore, inclusion of an independent researcher will help to ensure non‐biased interpretation of the data. To maintain transparency, a reflexivity statement will be included within the final report to document how the researchers’ roles, involvement with the group, preconceptions and viewpoints may have influenced the generation and analysis of data.^31^

## 3 DISCUSSION AND DISSEMINATION

‘The *Lancet Psychiatry* Commission on psychological treatments research in tomorrow's science' emphasized the vital importance of involving people with lived experience of suicide and self‐harm throughout all stages of the research process.^32^ Although there is useful guidance on involving the public in research,^33^, ^34^, ^35^ sensitive topics such as suicide and self‐harm present specific challenges for research teams. Furthermore, comprehensive reporting of the effects of PPI on the conducted research, PPI members and researchers, is crucial to the development of effective and meaningful PPI practice.^8^ Consequently, the planned study will seek to address these gaps in the literature by conducting a mixed‐methods, longitudinal evaluation of the effects that PPI in suicide and self‐harm research had on the research, the PPI members and the research team. Findings from this study will be reported in line with the GRIPP2 checklist^8^ and submitted for publication to a specialized peer‐reviewed journal. We will also provide a lay summary to all PPI members and researchers who participated in this evaluation. The need for discipline‐specific PPI guidance has previously been highlighted.^20^ Consequently, we will address this gap by utilizing the novel insights from this evaluation to inform and develop practical guidance for researchers working in the field of suicide and self‐harm research, who wish to involve people with experiential knowledge in their research. Further ideas for dissemination of findings will be developed in conjunction with member of the PPI panel.

Central to conducting ethical research is a commitment to ‘do no harm’.^36^ It is possible that PPI members involved in suicide, and self‐harm research may become distressed or upset when drawing upon their own personal experiences. Whilst distress does not necessarily constitute harm,^22^ it is important to examine both PPI members’ experiences in relation to distress,^20^ and also their views on the provision of appropriate support for PPI members in this context. In addition, in the event that PPI members become unwell, this may also impact the well‐being of researchers who have developed significant working relationships with PPI members. Consequently, it is also important to assess their views and well‐being in relation to facilitating PPI and providing support to panel members. This new knowledge will be utilized to develop good quality and safe practice for PPI in suicide and self‐harm research.

A key strength of the planned study is the mixed‐methods, longitudinal approach, which aims to utilize both qualitative and quantitative methods to enhance our understanding of PPI in suicide and self‐harm research.^37^ To minimize the likelihood of recall bias, we plan to use repeated assessment throughout the group's involvement, as opposed to an evaluation solely on completion of the project. The trustworthiness of the data will be enhanced through the use of multiple data collection methods (document reviews, questionnaires and interviews) and sources (PPI members and researchers).^31^ Finally, to enhance the quality and rigour of the qualitative analysis conducted, we will involve researchers who possess different expertise and backgrounds (ie PPI, qualitative, quantitative, suicide and self‐harm) and also include two independent researchers who have had no prior involvement with the PPI group. In conclusion, this study has important implications for research into sensitive topics such as self‐harm and suicide. Our robust methodological approach will generate new evidence‐based guidance for facilitating the enhanced involvement of people with experiential knowledge in suicide and self‐harm research. Increasing the level of meaningful involvement in self‐harm and suicide prevention research has the potential to transform current health service research in this area.

## CONFLICT OF INTEREST

NK is a member of the Department of Health's (England) National Suicide Prevention Advisory Group. He chaired the NICE guideline development group for the longer term management of self‐harm and the NICE Topic Expert Group (which developed the quality standards for self‐harm services). NK is currently chair of the updated NICE guideline for Depression and is also supported by the Greater Manchester Mental Health NHS Foundation Trust.

## Data Availability

The data that support the findings of this study are available from the corresponding author, DLL, upon reasonable request.
