# Supplementary material for: Evaluating the impact of patient and carer involvement in suicide and self‐harm research: A mixed‐methods, longitudinal study protocol
Source: Health Expect. 2019 Dec 5;24(Suppl 1):47–53. doi: 10.1111/hex.13000 (PMC8137496; doi:10.1111/hex.13000)
Supplement: Supplementary file 1 [file HEX-24-47-s001.docx]

**Overview of Patient and Public Involvement approach**

On initiation of the group’s involvement, we adhered to the PPI best practice principles as outlined by Baines, Regan de Bere ^18^. These principles were informed and defined on the basis of robust evidence generated by a systematic review and modified Delphi exercise. The principles cover communication, ways of working, and provision of practical, emotional and financial support.^18^ In accordance with INVOLVE guidance,^19^ we committed to reimburse members’ travel expenses, and provide compensation for attending group meetings and completing involvement tasks. Our approach was refined during the information session in which we discussed and agreed a series of ‘ground rules’ that would guide ways of working as a group (summarised in Table S1). The ground rules will be reviewed and revised periodically by the group, throughout the duration of the project.

**Table S1. Summary of PPI group co-developed ‘ground rules’**

| Communication |
| --- |
| - Researchers will maintain regular communication with PPI members, providing updates and feedback following their involvement in the research. |
| - Non-technical, plain English will be used and members will be given the opportunity to choose their preferred communication method - via email, post or telephone call. |
| Meetings |
| - Members will be provided with an agenda and any other relevant documents in good time in advance of meetings. |
| - The fee for attendance at meetings, or in relation to remote reviewing activities will be outlined prior to the involvement activity taking place, and arrangements for claiming travel expenses will be clarified by the researcher organising the meeting. |
| - Within the PPI meetings, people will treat each other with respect and work together as a team in seeking to reach general agreements. |
| General ways of working |
| - Involvement will be flexible, to allow members to opt-in and out of activities as they wish. |
| - When inviting members to complete an involvement task, researchers will provide an overview of the task, and specifically indicate: 1) the expected time commitment; 2) the deadline for its completion; 3) the fee for completing it. |
| - Matters discussed in relation to the group will be treated as confidential, including any personal information disclosed by members during meetings. Confidentiality would, however, need to be breached in the event that someone where to disclose information about risk of harm to self or to others. |
